# Systematic review of animal studies on the use of herbal medicine for attention-deficit/hyperactivity disorder

**DOI:** 10.3389/fpsyt.2026.1812398

**Published:** 2026-06-18

**Authors:** Gia Linh Mac, Khoa Nguyen Tran, Tien Dat Tran, Haifeng Shao, Yang Wang, Kyung-Hee Park, Hyo-Won Jung, In-Jun Yang

**Affiliations:** 1 Department of Physiology, Dongguk University College of Korean Medicine, Gyeongju, Republic of Korea; 2 Department of Herbal Medicine, Dongguk University College of Korean Medicine, Gyeongju, Republic of Korea

**Keywords:** ADHD, animal models, CAM, herbal medicine, preclinical studies

## Abstract

**Background:**

Attention-deficit/hyperactivity disorder (ADHD), a common neurodevelopmental condition, often requires treatment with stimulants such as methylphenidate and nonstimulants such as atomoxetine. Despite their effectiveness, medication-related side effects, adherence and symptom control issues have increased the interest in alternative therapies, especially herbal medicine. However, existing preclinical studies vary in quality and relevance. This systematic review evaluates animal studies investigating herbal medicines for ADHD; identifies limitations in experimental models, behavioral assessments, safety evaluation, and methodological rigor; and proposes a framework to guide future research.

**Methods:**

Following PRISMA 2020 guidelines, PubMed and Web of Science were systematically searched for eligible studies published between 1995 and March 2025 that used animal models to interventions for the treatment of ADHD-like symptoms.

**Results:**

Analysis of 25 studies revealed a strong reliance on a limited range of animal models, particularly the spontaneously hypertensive rat (48%), with 88% of studies using male animals exclusively. Behavioral assessments predominantly focused on hyperactivity (72%), whereas evaluations of inattention and impulsivity were comparatively limited. Herbal interventions were associated with improvements in ADHD-like behaviors and modulation of catecholaminergic neurotransmission, including increased dopamine and norepinephrine levels in the prefrontal cortex. Additional mechanisms included activation of neurotrophic signaling pathways (brain-derived neurotrophic factor/TrkB) and suppression of proinflammatory mediators such as TNF-α and IL-1β. However, 80% of the studies did not report safety outcomes, and no study clearly described randomization or blinding procedures, limiting the translational relevance of the findings. Despite these limitations, several formulations, including the Long Mu Qing Xin mixture, An Shen Ding Zhi Ling demonstrated promising multitarget effects.

**Conclusion:**

Although herbal medicines demonstrate promising multipathway effects for ADHD, progress remains constrained by the limited diversity of animal models and narrow scope of behavioral assessments. Future research should prioritize the use of diverse models, more comprehensive assessments, and the implementation of rigorous methodological standards.

## Graphical Abstract

**Figure:**
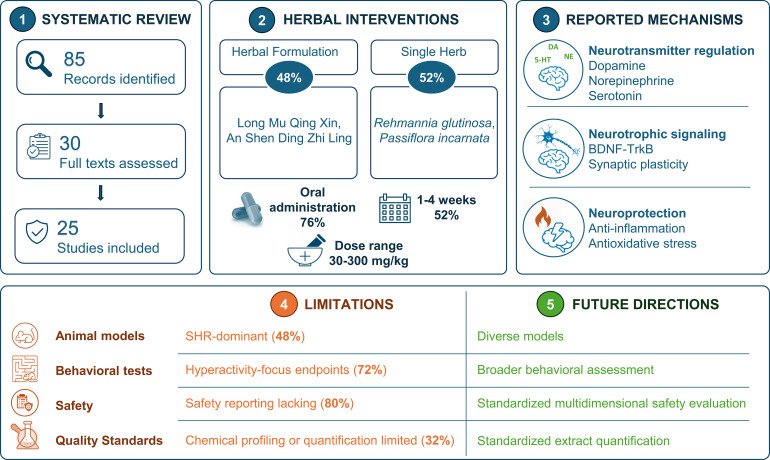

## 1 Introduction

Attention-deficit/hyperactivity disorder (ADHD) ranks among the most widespread neurodevelopmental disorders and affects approximately 8% of children and adolescents worldwide. It is characterized by persistent symptoms of inattention, hyperactivity, and impulsivity, which substantially impair daily functioning and development (1). Most ADHD studies have investigated children and adolescents, and evidence on adults remains limited because symptoms in adulthood are generally less apparent. Furthermore, increasing attention is being directed toward ADHD in the older adult population, with a prevalence of persistent ADHD reported at 2.58% in adults, affecting multiple domains of life, including educational attainment, employment, marital stability, and involvement in criminal behavior (2). However, the comprehensive understanding and effective management of ADHD in adults remain limited because of factors such as age, comorbid psychiatric conditions, and quality of life. Moreover, research on ADHD assessment, diagnosis, and treatment remains limited (3, 4).

Current primary treatment options include pharmaceutical, behavioral, and psychosocial components, either separately or in combination. Although stimulant medications such as methylphenidate (MPH) and nonstimulant agents such as atomoxetine (ATX) have demonstrated efficacy in managing ADHD symptoms, they are frequently associated with side effects such as appetite suppression, sleep disturbances, and increased anxiety (5). Additionally, concerns persist regarding long-term safety, treatment adherence challenges, and limited efficacy in certain patient subgroups.

These limitations have prompted a growing interest in complementary and alternative medicine interventions, including herbal medicines derived from traditional East Asian medical systems. Several preclinical studies have investigated the potential efficacy of herbal formulations in animal models of ADHD and reported improvements in hyperactivity, learning, and adaptive behavioral regulation. However, the quality, depth, and translational readiness of these studies vary substantially.

This systematic review aimed to critically evaluate the current landscape of herbal medicine research using animal models of ADHD and compare it with the evolving standards of modern preclinical neuroscience. Key limitations in model selection, behavioral assessment, and mechanistic analysis were systematically identified. The review further aimed to propose an integrative framework to advance herbal ADHD research in alignment with contemporary scientific rigor and translational expectations.

## 2 Methods

### 2.1 Databases and search terms

This systematic review followed the updated Preferred Reporting Items for Systematic Reviews and Meta-Analyses (PRISMA 2020) guidelines (6). Searches were conducted in PubMed and Web of Science for studies published from 1995 to March 2025 using the following search terms, with the syntax adapted to the requirements of each database: (“herbal medicine” OR “traditional Chinese medicine” OR “Korean medicine” OR “traditional medicine” OR “medicinal herb*” OR “herbal extract*” OR “natural product*” OR “plant extract*” OR “Chinese herb*” OR “medicinal plant*” OR “herbal preparation*” OR “herbal formula*” OR “herbal compound*” OR “herb*” OR “botanical*” OR “phytomedicine” OR “phytotherapy”) AND (“attention deficit disorder with hyperactivity”[MeSH] OR “ADHD”[Title/Abstract] OR “attention deficit hyperactivity disorder”[Title/Abstract] OR “hyperkinetic disorder”[Title/Abstract] OR “attention deficit”[Title/Abstract] OR “hyperactive behavior”[Title/Abstract] OR “hyperactivity disorder”[Title/Abstract]) AND (“animal model*” OR rat OR rats OR mice OR mouse OR rodent* OR “animal experiment*”) NOT (“clinical trial”[Publication Type] OR “human study”[Title/Abstract] OR “patient*”[Title] OR “clinical study”[Title/Abstract]) NOT (“review”[Publication Type] OR “review”[Title] OR “systematic review”[Title] OR “meta-analysis”[Publication Type] OR “meta-analysis”[Title] OR “literature review”[Title] OR “review of the literature”[Title] OR “overview”[Title]).

### 2.2 Eligibility criteria

Duplicates were removed from all retrieved records, followed by title and abstract screening. This review included original studies that investigated the therapeutic effects of herbal materials, including single herbs and multiherb formulations, on ADHD-like symptoms in animals. Clinical studies, cell-based experiments, review articles, and studies involving nonherbal interventions were excluded. Only studies published in English, Korean, or Chinese were retained for further analysis.

Full texts of the remaining articles were then accessed, and the following information was extracted: (1) publication year, (2) animal model, (3) herbal materials, (4) dosage, (5) comparator, (6) behavioral assessments, (7) underlying mechanisms, (8) side effects, and (9) references. Studies with inaccessible full texts were excluded. Data extraction was performed independently by four researchers, and disagreements were resolved through discussion with a fifth researcher until a consensus was reached.

### 2.3 Quality assessment method

The 2010 ARRIVE guidelines were used for quality assessment (7), according to criteria based on 20 items divided into 6 main groups: title, abstract, introduction, methods, results, and discussion. Each item was judged as “2,” “1,” or “0” equivalent to “clearly sufficient,” “possibly sufficient,” or “clearly insufficient”. Two researchers independently conducted this process, and a third researcher resolved disagreements through discussion until a consensus was reached.

## 3 Results and discussion

### 3.1 Selection results and quality assessment

**Figure 1:**
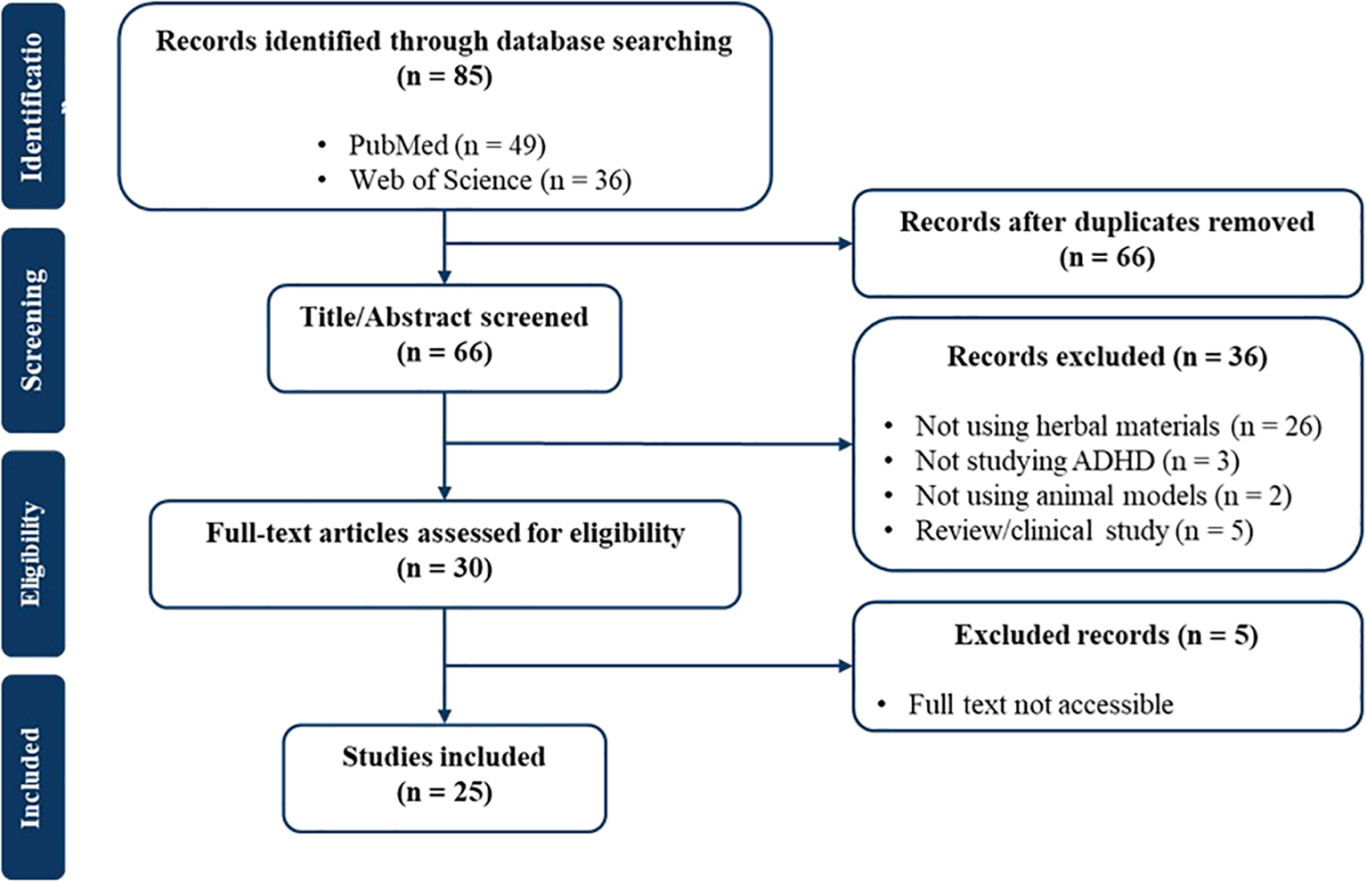
Flowchart of study selection.

**Figure 1** illustrates the study selection process for this review. The initial search identified 85 records, including 49 from PubMed and 36 from Web of Science. After excluding 19 duplicates, 66 records remained for title and abstract screening, of which 36 were excluded. The full texts of the remaining 30 studies were assessed for eligibility, and 25 studies ultimately met the inclusion criteria.

**Tables 1**, **2** summarize the characteristics of the 25 included studies. Only a few studies systematically reported adverse effects; gastric irritation and impaired weight gain were observed in some studies, whereas most studies reported no notable toxicity. Collectively, the evidence suggests that herbal medicines may alleviate hyperactivity, cognitive deficits, and emotional regulation in ADHD-like models through multiple target mechanisms, including neurotransmitter modulation, synaptic plasticity enhancement, anti-inflammatory and antioxidant effects, and neurodevelopmental regulation.

**Table 1:** Summary of studies using herbal formulations.

No.	Publication year	Animal model	Herbal materials	Dose/duration	Comparative study	Behavioral assessment	Mechanisms	Side effects/Adverse effects reported	Reference
1	1995	Scopolamine-treated Swiss mice	Tiaoshen Liquor (TL)	3000, 6000 mg/kg 3 days	NR	Step-down test	**Cholinergic neurotransmission**	NR	(8)
2	2024	SHR	Long Mu Qing Xin mixture (LMQXM)	5.28, 10.56, and 21.12 mL/kg/day 4 weeks	MPH	OFT and MWM	**Catecholaminergic neurotransmission:** DA, NE; DRD1/cAMP/PKA-CREB	Gastric mucosa irritation at high doses	(9)
3	2024	SHR	Jieyu pills	1080, 2160, and, 4320 mg/kg/day single dosing	NR	NR	NR	NR	(10)
4	2023	SHR	Long Mu Qing Xin mixture (LMQXM)	22000, 44000, and, 88000 mg/kg 4 weeks	MPH	OFT and MWM	**Dopaminergic signaling:** DRD1-mediated cAMP/PKA signaling	NR	(11)
5	2022	SHR	Jing-Ning granules (JNG)	5785, 11570, and, 23140 mg/kg 6 weeks	ATX	OFT and Step-down test	**Dopaminergic receptor signaling:** D1/D2-like receptor-mediated cAMP/PKA and Ca²^+^/CaM/CaMKII signaling	NR	(12)
6	2022	SHR	An Shen Ding Zhi Ling (ASDZL)	27400 mg/kg 4 weeks	MPH	OFT and MWM	**Neurotrophic signaling / dopamine vesicle regulation:** BDNF/TrkB and BDNF/p75/JNK1/NF-κB	NR	(13)
7	2022	SHR	Dimu Ningshen (DMNS)	4.05 mg/kg 22 weeks, including short-term (6 weeks) and long-term (13 weeks)	MPH	OFT Y-maze, NORT, and 5-CSRTT	**Gut–brain axis / metabolomics:** gut microbiota and circulating peripheral metabolites	No significant side effects were observed in liver, kidney, and heart functions.	(14)
8	2021	SHR	An Shen Ding Zhi Ling (ASDZL)	21250 mg/kg 4 weeks	ATX	OFT and MWM	**Neuroinflammation and BBB integrity:** cytokines, glial markers, and BBB protection	No significant side effects were observed in animal weight changes.	(15)
9	2020	Prenatal alcohol-exposed mice	HX106	200 mg/kg/day 3 weeks	NR	OFT and Y-maze	**Dopamine signaling and metabolomics:** DAT, DRD2; plasma/liver/colon/feces metabolite profiles	NR	(16)
10	2017	Social isolation stress model in ICR mice	Sansoninto (SST)	800 and, 2400 mg/kg 7 weeks	NR	Sociability test, water-finding test, and fear-conditioning test	**Cholinergic-related signaling:** Egr-1	NR	(17)
11	2017	Social isolation stress model in ICR mice	Yokukansan (YKS), Keishito (KST)	YKS, 1523.6 mg/kg and KST, 2031.8 mg/kg 8 weeks	NR	3-chamber test, water-finding test, and fear-conditioning test	**Dopaminergic/cholinergic neuroplasticity signaling:** CaMKII and CREB	NR	(18)
12	2014	Aroclor, 1254-treated ICR mice	YY162	200 mg/kg 14 days (PND 21–35)	MPH	OFT, object-based attention test, and EPM	**Oxidative stress and monoamine transporter regulation:** ROS, MDA, protein carbonyl; DAT/NET recovery associated with BDNF signaling	NR	(19)

5-CSRTT, Five-choice serial reaction time task; ADHD, Attention-deficit/hyperactivity disorder; ATX, Atomoxetine; BBB, Blood–brain barrier; BDNF, Brain-derived neurotrophic factor; DA, Dopamine; DAT, Dopamine transporter; DRD2, D2 dopamine receptor; Egr-1, Early growth response 1; EPM, Elevated plus maze; GFAP, Glial fibrillary acid protein; Iba1, Ionized calcium binding adaptor molecule 1; IL-10, Interleukin-10; IL-1β, Interleukin-1β; IL-4, Interleukin-4; IL-6, Interleukin-6; JNK1, C-Jun N-terminal kinases 1; MAPK, Mitogen-activated protein kinase; MCP-1, Monocyte chemoattractant protein 1; MDA, Malondialdehyde; MPH, Methylphenidate; MWM, Morris water maze; NE, Norepinephrine; NET, Norepinephrine transporter; NF-κB, Nuclear factor kappa B; NORT, Novel object recognition test; OFT, Open field test; p75, p75 neurotrophin receptor; ROS, Reactive oxygen species; SHR, Spontaneously hypertensive rat; TNF-α, Tumor necrosis factor-α; TrkB, Tyrosine kinase receptor B.

**Table 2:** Summary of studies using single herbs.

No.	Publication year	Animal model	Herbal materials	Dose/duration	Comparative study	Behavioral assessment	Mechanisms	Side effects/Adverse effects reported	Reference
1	2016	Normal Wistar Albino Glaxo rats	*Passiflora incarnata* L.	30, 100, 300 mg/kg/day 7 weeks	NR	Modified MWM, visible platform test, and defecation count	**Monoaminergic and GABAergic neurotransmission:** 5-HT, NA, GABA, 5-HIAA/5-HT ratio, and DOPAC/DA ratio.	NR	(20)
2	2024	DAT-knockdown mice	Rhynchophylline (RHY)	30 mg/kg 8 weeks	NR	OFT and MWM	**Neuroinflammation:** TNF-α, IL-1β, iNOS, COX-2	NR	(21)
3	2024	SHR	Rehmanniae Radix Preparata (RRP)	2400 mg/kg 4 weeks	MPH	OFT, MWM, and EPM	**Neurogenesis and synaptic plasticity:** TrkB, CDK5, FGF/FGFR	NR	(22)
4	2023	Valproic acid-treated rats	*Passiflora incarnata* L.	30, 100, 300 mg/kg 46 days	NR	OFT, EPM, NORT, three-chamber test, and marble burying test	**Oxidative stress and histopathological neuroprotection:** MDA, SOD, CAT, TAC; neuronal damage markers	NR	(23)
5	2022	SHR	Rehmanniae Radix Praeparata (RRP)	2400 mg/kg 4 weeks	MPH	OFT	**Mitochondrial and energy metabolism:** mitochondrial respiratory function, SDH, COX, ATPases, mPTP, GLUT1, GLUT3	No significant side effects were observed in animal weight changes.	(24)
6	2021	Neonatal habenula lesion rats	*Ecklonia stolonifera* (ES), fucosterol (FCS)	ES: 100, 200 mg/kg FCS: 1 mg/kg postnatal days 21–27	NR	Locomotion test; Delay discounting test; Object exploration test	**Neuroelectrophysiological signaling:** restoring behavioral functions by reducing excessive LFP signaling synchronization between AMY and PFC.	NR	(25)
7	2020	AlCl_3_-treated BALB/c mice	*Rosmarinus officinalis*	100 mg/kg 20 days	MPH	MWM and Y-maze	**Synaptic plasticity and neuroinflammation:** neuronal density, Synapsin I–III, APP770; TNF-α, IL-6, GFAP	NR	(26)
8	2019	SHR	Catalpol	50 mg/kg/day 4 weeks	MPH	OFT and MWM	**Neurodevelopmental signaling:** BDNF, Cdk5/p35, FGF21/FGFR1	NR	(27)
9	2020	Normal ddY mice	*Lantana camara*	0.000004, 0.00004, 0.0004, 0.004, 0.04, and 0.4 mg (per 400 μL of triethyl citrate, TEC)	NR	OFT	NR	No side effects such as increased urination or defecation were observed during the experiments.	(28)
10	2018	SHR	Shudihuang	2400 mg/kg/day 4 weeks	MPH	OFT	**Neurodevelopmental/synaptic plasticity signaling:** BDNF, TrkB, NRG-3	NR	(29)
11	2007	Cocain treated C57BL/6 mice	Fructus Psoraleae (FPS)	20, 100, and 500 mg/kg	NR	Locomotor activity tests	**Monoaminergic transporter regulation:** DA/DAT and NE/NET	NR. However, FP was observed to increase locomotor activity without immediate toxicity in behavioral tests.	(30)
12	2008	SHR	Oroxylin A	2 mg/kg, 10 mg/kg	MPH	Locomotor activity test, EPM, and Rota-rod test	**GABAergic neurotransmission:** GABA_A receptor-mediated modulation	NR	(31)
13	2014	Normal Wistar albino rats	*Catha edulis*	500 mg/kg 23 days	MPH	Radial arm maze	**Catecholaminergic neurotransmission:** dopaminergic and noradrenergic systems	NR	(32)

5-HIAA, 5-hydroxyindoleacetic acid; 5-HT, Serotonin; BDNF, Brain-derived neurotrophic factor; CAT, Catalase; CDK5, Cyclin-dependent kinase 5; COX, Cytochrome C oxidase; COX-2, Cyclooxygenase-2; DA, Dopamine; DAT, Dopamine transporter; DAT-, Dopamine transporter knockdown; DOPAC, 3,4-dihydroxy-phenylacetic acid; EPM, Elevated plus maze; FGF, Fibroblast growth factor; FGFR, Fibroblast growth factor receptor; GABA, Gamma-aminobutyric acid; GFAP, Glial fibrillary acid protein; GLUT1, Glucose transporter 1; GLUT3, Glucose transporter 3; IL-1β, Interleukin-1β; IL-6, Interleukin-6; iNOS, inducible nitric oxide synthase; MDA, Malondialdehyde; MPH, Methylphenidate; mPTP, mitochondrial permeability transition pore; MWM, Morris water maze; NA, Noradrenaline; NE, Norepinephrine; NET, Norepinephrine transporter; NORT, Novel object recognition test; OFT, Open field test; PFC, Prefrontal cortex; RAM, Radial arm maze; SDH, Succinate dehydrogenase; SHR, Spontaneously hypertensive rat; SOD, Superoxide dismutase; TAC, Total antioxidant capacity; TNF-α, Tumor necrosis factor-α; TrkB, Tyrosine kinase receptor B.

The comprehensive assessment of reporting quality across the 25 studies identified several strengths and important limitations that should be addressed (**Table 3**). All 25 studies provided concise titles, executive summaries, clearly defined objectives, and identifiable experimental outcomes, indicating generally well-structured and coherent reporting. Additionally, most studies adequately described the scientific context and presented findings clearly, supporting transparency. However, systematic evaluation using the ARRIVE guidelines identified important limitations in reporting adverse effects, study design, and clinical translation that should be addressed. Although most studies stated that animals were “randomly divided,” none adequately described specific randomization procedures or allocation concealment during treatment and behavioral assessment. Consequently, no study was rated “clearly sufficient” in these categories, raising concerns regarding reproducibility. This lack of transparency extended to methodological detail, with only 44% of studies providing comprehensive procedural descriptions and 36% reporting complete animal characteristics. Key information including genetic status, rearing conditions (e.g., light–dark cycles), and the rationale for herbal formulations was frequently omitted, limiting cross-study comparability.

**Table 3:** Quality assessment of the included studies.

Reference	Items																			
	1	2	3	4	5	6	7	8	9	10	11	12	13	14	15	16	17	18	19	20
8	2	2	1	2	0	1	1	1	0	1	1	2	1	1	2	2	0	1	1	0
20	2	2	1	2	2	1	2	1	1	1	1	2	1	0	2	2	0	1	1	1
21	2	2	2	2	2	1	2	2	1	1	1	2	2	1	2	2	0	1	1	2
9	2	2	2	2	2	1	1	2	1	1	1	2	1	1	2	2	1	1	1	2
22	2	2	2	2	2	1	2	1	1	1	2	2	1	1	1	2	0	1	1	2
10	2	2	2	2	2	1	2	2	1	1	0	2	1	2	1	1	0	1	1	2
23	2	2	2	2	2	1	1	2	1	1	1	2	1	1	2	2	0	1	1	2
11	2	2	2	2	2	1	1	2	1	2	1	2	2	2	2	2	1	2	1	2
12	2	2	2	2	2	1	1	2	1	1	1	2	1	1	2	2	0	1	1	2
13	2	2	2	2	2	1	2	2	1	1	1	2	1	1	2	1	0	1	1	2
24	2	2	2	2	0	1	1	2	1	1	1	2	1	1	2	2	0	2	1	2
14	2	2	2	2	2	1	1	1	1	1	1	2	1	1	1	2	0	1	1	2
2	2	2	2	2	2	1	1	1	1	1	1	2	1	2	1	2	0	1	1	2
25	2	2	2	2	2	1	1	1	1	1	1	2	1	1	2	2	0	1	1	2
16	2	2	2	2	2	1	2	2	1	1	0	2	2	1	2	2	0	1	1	2
26	2	2	2	2	2	1	2	1	1	1	0	2	1	0	2	2	0	1	1	2
27	2	2	2	2	2	1	2	1	1	1	1	2	1	1	2	2	0	1	1	2
28	2	2	2	2	2	1	2	1	1	1	0	2	1	0	2	2	1	1	1	2
29	2	2	2	2	0	1	2	1	1	1	1	2	1	2	2	2	0	1	1	1
17	2	2	2	2	2	1	1	1	1	1	0	2	1	0	1	2	1	1	1	2
18	2	2	2	2	2	1	1	1	1	1	1	2	1	0	2	2	1	1	1	2
30	2	2	2	2	1	1	2	1	1	1	1	2	1	0	2	2	0	1	1	2
31	2	2	2	2	1	1	1	1	1	1	1	2	1	1	1	2	0	1	1	1
32	2	2	2	2	1	1	1	1	0	1	1	2	1	0	2	2	0	1	1	0
19	2	2	2	2	1	1	1	1	1	1	1	2	1	0	2	2	0	1	1	1
The percentage of “2”(%)	100	100	92	100	72	0	44	36	0	8	4	100	12	16	76	92	0	8	0	76
The percentage of “1”(%)	0	0	8	0	16	100	56	64	92	92	76	0	88	52	24	8	20	92	100	16
The percentage of “0”(%)	0	0	0	0	12	0	0	0	8	0	20	0	0	32	0	0	80	0	0	8

“2”, clearly sufficient; “1”, possibly sufficient; “0”, clearly insufficient.

Furthermore, statistical and safety reporting was found to be inadequate. Notably, 80% of the studies did not report major adverse events, suggesting potential reporting bias and underreporting of negative or unexpected outcomes. A substantial gap in translational validity was observed. Despite reporting neurochemical findings, most studies did not relate these findings to human ADHD pathophysiology or address the limitations of the selected animal models. To bridge the gap between basic neuroscience and clinical application, future studies should provide in-depth discussion on how model validity affects the potential effectiveness of herbal therapies in humans. The inclusion of a dedicated “Safety Evaluation” section and the use of standardized human equivalent dose calculations will further strengthen the progression toward a more robust, evidence-based clinical framework.

### 3.2 ADHD animal models

A key determinant of successful preclinical ADHD research is the selection of an animal model that accurately reflects the core features of the disorder. Among the 25 articles included, nearly half (n = 12; 48%) employed the classical spontaneously hypertensive rat (SHR) model. The remaining studies employed various models based on genetic or neurodevelopmental alterations, including dopamine transporter (DAT)-knockdown mice (two studies) and neonatal habenula lesion (NHL) rats (two studies). Additionally, five studies (20%) employed environmentally induced ADHD models, including chemical exposures such as prenatal alcohol exposure (PAE) (one study); valproic acid (VPA)-induced autism (one study); aluminum chloride exposure (one study); and Aroclor 1254, dl-methylphenidate, or GBR12909 exposure (one study), as well as psychosocial stress (social isolation [SI]). Collectively, these models reflect diverse aspects of ADHD pathophysiology, including dopaminergic dysregulation, impaired synaptic plasticity, and altered developmental stress responses.

Currently, consensus across the scientific community highlights three essential dimensions of model validity: face validity (symptom resemblance), construct validity (etiological relevance), and predictive validity (treatment responsiveness) (33). Although the SHR model demonstrates strong face validity for hyperactivity, it does not adequately represent core features, such as sustained inattention and impulsivity (34). The lack of full symptom expression may exaggerate treatment effectiveness and limit the broad applicability of the study. The recent Long Mu Qing Xin mixture (LMQXM) study used SHR/NCrl rats, which serve as an optimal experimental model for the combined subtype of ADHD by satisfying the three fundamental criteria for animal model validation. First, the model demonstrates face validity, as evidenced by the overt manifestation of hyperactivity and significant impairments in innate learning and memory. Second, it possesses construct validity, characterized by a profound deficiency in catecholamine neurotransmitters (dopamine and norepinephrine), alongside the disruption of the DRD1/cAMP/PKA-CREB signaling pathway within the prefrontal cortex and striatum. Finally, the model exhibits predictive validity, as both the positive control methylphenidate and the LMQXM formula effectively mitigated behavioral abnormalities and successfully reversed the underlying neuropathological deficits, thereby confirming the therapeutic potential of the remedy within a scientifically rigorous framework. However, the experimental validity of such models is linked to the selection of an appropriate control group. The WKY/NCrl substrain, although frequently utilized in existing literature, is increasingly considered unsuitable as a control due to its high genetic similarity (76.6%) to the SHR/NCrl model and its inherent behavioral deficits, including inattention and anxiety. Conversely, WKY/NHsd is the optimal control for SHR/NCrl research. Its lower genetic homology (66.5%) and stable cognitive profile provide a clear, healthy baseline, allowing for a precise evaluation of behavioral and molecular differences. Therefore, to enhance the genetic and behavioral differences between experimental groups, future studies should employ the SHR/NCrl model in combination with the WKY/NHsd rat group as definitive control (9).

The DAT-knockdown mouse model targets core ADHD pathogenesis by disrupting the key regulator of dopaminergic signaling and shows high apparent validity. However, this is a single-gene model, whereas human ADHD is a complex, multigenic, multifactorial disorder (21). In contrast, the NHL model induces behavioral changes similar to ADHD symptoms in humans (hyperactivity, impulsivity, and inattention) that are highly responsive to standard stimulants such as amphetamine. Although this finding demonstrates strong predictive validity, the model is limited by the age-dependent nature of its symptoms, where hyperactivity often reduces as the subjects reach adulthood (25).

In addition to genetic interventions, environmental and neurotoxic models provide a broader perspective on developmental risks. The SI model, which involves isolating pups after weaning, produces an environmentally induced phenotype characterized by increased reactivity and impaired social interaction. It serves as a potential animal model of a developmental disorder with comorbid features of ADHD and autism spectrum disorder (ASD), although its utility remains limited by the absence of intrinsic genetic factors (17, 18). Similarly, prenatal chemical exposure models using VPA or PAE demonstrate how early-life neurotoxic insults can lead to persistent hyperactivity and inattention in offspring. Although the VPA model is particularly useful for simulating the symptomatic overlap between ADHD and ASD, it has key limitations in practical translation (23). Models involving exposure to Aroclor 1254, scopolamine, and AlCl_3_ were effective in producing models of impaired learning ability, spatial memory, and concentration. However, they tended to focus on inducing memory impairment, making them more suitable for studies on Alzheimer’s disease or dementia (26).

Research on herbal remedies for ADHD remains largely based on a limited set of animal models, constraining its integration within a broader translational framework. To address this limitation, future studies should diversify the range of animal models by combining genetic and environmental paradigms. The oroxylin A study also accurately discussed ADHD subtypes based on three core symptoms. The study reported that ADHD is manifested through persistent hyperactivity, impulsivity, and inattention, categorized into three subtypes: primarily inattentive, primarily hyperactive/impulsive, and combined in type (31). Therefore, such a combined approach would more comprehensively reflect the heterogeneity of ADHD, enabling the selection of herbal compounds tailored to symptom clusters based on bioinformatics data and ultimately enhancing the specificity, scalability, and clinical relevance of preclinical findings.

### 3.3 Behavioral evaluation tools: scope and gaps

Behavioral tests were used to replicate core ADHD manifestations, including hyperactivity, inattention, and impulsiveness, providing a robust framework for evaluating therapeutic efficacy. An analysis of 25 selected studies revealed a significant methodological bias: 18 studies (72%) focused on assessing hyperactivity. Within this subset, the open field test (OFT) accounted for 83.3% of the behavioral assessments, followed by the Morris water maze test at 40%, with nine studies focusing on spatial learning and memory. Additionally, Y-maze, step-down, and novel object recognition tests were used to assess learning and memory abilities. These data indicate a disproportionate emphasis on hyperactivity while neglecting the core symptoms of inattention and impulsivity, which significantly impair daily functioning in patients and remain markedly underrepresented.

This narrow focus limits the scope of research to purely motor functions and general cognition rather than a comprehensive assessment of ADHD pathophysiology. Furthermore, the infrequent application of cognitive-specific paradigms, such as the five-choice serial reaction time task (5-CSRTT), delay discounting, and object exploration tests, along with the limited assessment of comorbid affective dysregulation markedly diminishes the face validity of these models. This disconnect suggests that current experimental frameworks fail to adequately capture the primary clinical manifestations of the disorder.

Furthermore, the interpretation of behavioral outcomes presents inherent logical challenges. Relying solely on reduced locomotor activity in the OFT as evidence of efficacy impedes the differentiation of genuine therapeutic benefits from nonspecific sedation or general arousal modulation. Without metrics for attention or inhibitory control, the underlying pharmacological mechanisms remain unclear. This limited behavioral assessment acts as a translational barrier that hinders the clinical application of herbal agents.

To overcome these limitations, future studies should implement multidimensional behavioral batteries capable of capturing the full ADHD symptomatic spectrum. These behavioral findings should be integrated with neuroanatomical changes and pharmacokinetic data to provide a more rigorous scientific basis for therapeutic evaluation.

### 3.4 Herbal intervention characteristics and underlying mechanisms

Of the 25 studies, herbal formulations accounted for 48% (12 studies), whereas the remaining 52% (13 studies) focused on single herbal materials. Herbal interventions ranged from single extracts and compounds to multiherb formulations. Among the herbal formulations, *Glycyrrhiza* (6 studies) was the most frequently investigated, followed by *Uncariae Ramulus cum Uncis* (5 studies), *Angelica sinensis* (5 studies), *Rehmannia glutinosa*, and *Schisandra chinensis* (4 studies). Other herbs, such as *Scutellaria baicalensis*, *Bupleurum*, *Paeonia lactiflora*, and *Ziziphus jujuba* Mill. were examined in four studies. Within the single-herb category, *Rehmannia glutinosa* was reported most frequently (4 studies), followed by *Passiflora incarnata* (2 studies). Overall, *Rehmannia glutinosa* was the most frequently studied herbal material in both categories (7 studies).

Oral administration was the predominant route in 19 studies (76.0%), injection was used in 5 studies (20.0%), and inhalation was used in 1 study (4.0%). Regarding treatment duration, 13 studies (52.0%) were conducted for 1–4 weeks, 5 (20.0%) lasted for 4–8 weeks, 3 (12.0%) extended beyond 8 weeks, and 4 (16.0%) did not report treatment duration. The dosage of orally administered herbal extracts generally ranged between 100 and 300 mg/kg/day, although wider variations were observed (mouse: 30–2400 mg/kg/day, rat: 30–88,000 mg/kg/day) depending on the specific material or formulation, extraction method, or animal model.

Mechanistic studies most commonly focused on three core signaling systems: monoaminergic neurotransmitter (dopamine, norepinephrine, and serotonin), neurotrophic signaling (brain-derived neurotrophic factor [BDNF]/TrkB and FGF/FGFR), and anti-inflammatory (IL-1β, TNF-α, and NF-κB) and oxidative stress pathways (malondialdehyde, superoxide dismutase [SOD], catalase [CAT], and total antioxidant capacity [TAC]). Additional mechanisms included modulation of cellular bioenergetics, including Na^+^/K^+^-ATPase activity and mitochondrial function. The primary mechanism identified in 40% of studies involves the regulation of neurotransmitter systems, chiefly dopaminergic pathways (including D1 signaling and DAT function) and, to a lesser extent, noradrenergic and cholinergic pathways (21.7%). For instance, *Fructus Psoraleae* extract optimizes dopamine (DA) and noradrenaline (NE) levels by directly modulating transporters (DAT and NET) and reuptake processes (30). This effect is complemented by the stabilization of the GABAergic system, where compounds such as oroxylin A and *Passiflora* (Passionflower) extracts regulate GABAA receptors and Cl-ion currents to suppress impulsivity (31). *Passiflora* further modulates synaptic activity by reducing excitatory neurotransmitter levels, including glutamic acid and serotonin, thereby restoring the neurochemical equilibrium essential for attention regulation (20).

Additionally, 36% of the studies highlighted the activation of neurotrophic pathways (cAMP/PKA/CREB, BDNF/TrkB, and FGF/FGFR), which drive synaptic plasticity and learning. Active compounds such as catalpol and rosemary enhance the expression of BDNF and its receptor TrkB. This upregulates synaptic proteins such as synapsin, driving neurogenesis and synaptic plasticity to reverse developmental memory deficits (26, 27).

Finally, 24% of the studies reported a neuroprotective role involving the inhibition of inflammatory mediators (TNF-α, IL-1β, and COX-2) and enhancement of antioxidant capacity (SOD, CAT, and TAC) to optimize the neural environment. Active compounds such as rhynchophylline from *Uncaria rhynchophylla* and rosemary extract inhibit glial cell overactivation (microglia and astrocytes) and substantially downregulate inflammatory markers such as TNF-α, IL-1β, and GFAP (21, 26). At a deeper cellular level, these natural agents modulate mitochondrial bioenergetics and intracellular signaling pathways. For example, catalpol restores mitochondrial function and optimizes ATP levels in the prefrontal cortex while regulating the Cdk5/p35 and FGF21/FGFR1 pathways to preserve neuronal spike density (27). The stability of neural networks is further refined by fucosterol, which normalizes abnormal local field potential oscillations in the amygdala–prefrontal cortex axis (25). Even the olfactory system may serve as a therapeutic gateway, with volatile monoterpenes in *Lantana camara* essential oil exerting rapid anesthetic and modulatory effects on the central nervous system (28).

The clinical superiority of complex herbal formulations such as Tiaoshen, LMQXM, and An Shen Ding Zhi Ling (ASDZL) lies in their synergistic, “push–pull” pharmacodynamics. These formulations target a single receptor and restore the balance between excitatory D1 and inhibitory D2 receptors through the cAMP/PKA-CREB and Ca^2+^/CaM/CaMKII signaling cascades. For example, BDNF axis modulation simultaneously activates prosurvival TrkB pathways and inhibits the antagonistic p75/JNK1/NF-κB inflammatory pathway (8, 11, 13 and 9). Furthermore, these polyherbal strategies leverage the gut–brain axis to increase peripheral monoamine precursors and optimize pharmacokinetic profiles. Compounds such as Z-ligustilide enhance blood–brain barrier permeability, whereas others facilitate the biotransformation of prodrugs into their active metabolites, such as liquiritin to liquiritigenin (10). This multilayered approach enables maximum therapeutic efficacy at moderate doses, maintaining systemic homeostasis and minimizing the toxicity often associated with high-dose, single-target synthetic pharmaceuticals.

Mechanistic analysis revealed similarities between the two approaches, which interfere with the three main pathophysiological signaling systems implicated in ADHD. The key difference between the mechanisms of individual herbs and combination remedies is that individual herbs typically act on specific biological targets, whereas combination remedies tend to produce synergistic, multitarget, and systemic effects. The central findings of neurotransmitter regulation are highly consistent with established ADHD neurobiology, in which dopaminergic and noradrenergic dysregulation are the core pathophysiological features (35, 36). Although there is a mechanistic overlap with FDA-approved drugs such as ATX in the modulation of dopaminergic (D1 and DAT) and noradrenergic (NET) systems (37), botanical interventions such as LMQXM and YY162 extend their effects to include neurotrophic signaling pathways (13, 19). Notably, this finding aligns with those of recent reports demonstrating that the benefits of nonpharmacological interventions, such as exercise or enriched environments, arise through neurotrophic pathways in ADHD models, highlighting a broader therapeutic axis that remains underutilized in current clinical practice (38, 39). Furthermore, by mitigating oxidative stress and neuroinflammation (e.g., TNF-α and IL-1β), herbal compounds optimize the cellular environment for synaptic remodeling, effectively reversing the inflammation-induced suppression of BDNF (40). Finally, although less extensively studied, the potential of herbal agents to enhance GABAergic tone represents a promising therapeutic strategy. ADHD has traditionally been conceptualized as a disorder of cortical excitatory–inhibitory imbalance, in which excessive glutamatergic activity and insufficient inhibitory tone contribute to hyperactivity and attentional deficits (41, 42). GABA receptors play a central role in inhibitory signaling in the brain, and deficits in GABAergic function have been reported in clinical and preclinical studies of ADHD (41, 43).

### 3.5 Comparative studies with standard therapies

The most common limitation across the current body of studies is the lack of direct comparisons with established pharmacological treatments. This limitation has been frequently acknowledged in the literature. Some studies compared herbal formulations with MPH (12 studies) or ATX (2 studies) using standardized behavioral paradigms. However, these comparisons are often compromised by unblinded designs, inconsistent dosing durations, or incomplete statistical analyses, thereby limiting the translational interpretation of efficacy claims. In addition, most studies lack key methodological safeguards, including randomization, dose matching, and blinded outcome assessment, which may introduce bias and complicate data interpretation. Furthermore, the lack of standardized outcome measures and effect size reporting restricts the generalizability of the findings. Consequently, in the absence of robust head-to-head comparisons with first-line stimulant therapies, current mechanistic and efficacy conclusions regarding herbal interventions should be considered preliminary and hypothesis-generating.

To enhance the clinical applicability of herbal medicine research in ADHD, future studies should prioritize comparative trials with validated positive-control drugs. Such studies should incorporate equivalent dose adjustments, parallel behavioral and molecular endpoints, and blinded outcome assessment. Comparative pharmacological studies are essential to establish the relative efficacy and translational relevance of herbal candidates.

In addition, bridging the gap between preclinical findings and clinical application requires improved dose standardization. Therefore, researchers should consider human equivalent dose calculations based on body surface area. For example, one study applied the approach described in the Methodology of Pharmacological Research of Traditional Chinese Medicines to convert the concentrations of *Rehmanniae Radix Preparata* between mice and humans (22). Similarly, studies on the LMQXM formula have calculated dosages in animals using the Stevenson formula (for children) and the Meeh–Rubner formula (for mice). Other studies, including those on ASDZL and Jing-Ning granules, also reported equivalent dose conversion approaches based on the second edition of Experimental Methods of Pharmacology (1991) (12, 13).

### 3.6 Safety evaluation, growth effects, and comorbid conditions

The long-term utility of any therapeutic strategy for ADHD, whether herbal or otherwise, depends on its efficacy, safety profile, developmental impact, and ability to manage comorbid symptoms. Studies on herbal-based ADHD treatment show substantial deficiencies in these domains.

First, preclinical safety assessments in herbal ADHD research remain sporadic and mainly qualitative, lacking the necessary empirical evidence to substantiate safety claims. Our evaluation revealed that 80% of the included studies failed to document adverse events systematically, frequently relying on rudimentary observations such as body weight fluctuations or reduced food intake. Notably, “gold-standard” biochemical markers, specifically alanine aminotransferase and creatinine, were reported in a single investigation (14). Furthermore, histopathological evaluations to assess structural damage were rare and conducted in only 28% of the investigations (12, 14, 22, 23, 25–27). Additionally, pharmacokinetic profiling was largely absent, with the sole exception of one study (10).

Second, the developmental implications of chronic herbal medicine administration remain poorly characterized. ADHD typically emerges early in life and often requires long-term treatment; however, most studies neither stratified outcomes by developmental stage nor assessed longitudinal effects on neuroendocrine maturation, sexual development, or physical growth. Analysis of animal characteristics revealed a pronounced sex bias, with 88% (22/25) of studies exclusively using male rodents and only 12% including both sexes. Experimental models were also largely restricted to pediatric stages; 16 studies used rodents aged 3–4 weeks, and 4 studies targeted the 6–8 weeks range. Notably, 16% (4/25) of the studies failed to report animal age, indicating a breach of the ARRIVE reporting standards. This limited representation of sex and developmental diversity, combined with inconsistent age reporting, constrains the interpretation of developmental stage–dependent efficacy and reduces the reproducibility and generalizability of pharmacological findings. Only a few studies reported neutral effects on body weight, and no study evaluated potential endocrine disruption or delayed maturation.

Third, the role of herbal therapies in modulating comorbid symptoms, such as anxiety, depression, sleep disturbances, and learning disabilities, remains largely insufficient. Considering the high comorbidity burden in patients with ADHD, this omission undermines the translational value of the current literature. Pharmaceutical studies are increasingly incorporating comorbid features into behavioral paradigms, and herbal studies should adopt this approach.

To address these gaps, future investigations should adopt a standardized toxicology framework that mandates quantitative biochemical monitoring of hepatic and renal biomarkers, systematic histopathologic assessment of target brain regions such as the prefrontal cortex and striatum, and integrated pharmacokinetic analyses to establish clear safety thresholds before conducting human trials. Furthermore, sex-based analyses should be integrated into experimental designs to evaluate potential sex-specific effects, an area that remains largely unaddressed in current studies.

In summary, although the absence of major reported toxicities in short-term studies is encouraging, the lack of rigorous multidimensional safety assessments constitutes a significant barrier to clinical translation. Bridging this gap requires comprehensive experimental frameworks that integrate efficacy, safety, and developmental outcomes within the same study design.

### 3.7 Herbal medicine standardization and quality control

Despite the pharmacological importance of active compounds, our analysis revealed that only 32% (8/25) of the included studies performed specific chemical profiling or quantification of active ingredients. To ensure the reproducibility and translational value of preclinical findings, rigorous standardization and quality control protocols are essential. However, the current literature exhibits considerable variability in these domains. Future studies must prioritize the standardization of extract preparation methods, specifically by providing detailed information on solvent types, extraction ratios, temperature, and duration, as these parameters critically influence the bioactive profile of the final product. Furthermore, comprehensive identification and quantification of active ingredients are required to move beyond descriptive analysis. Using advanced techniques such as ultra-performance liquid chromatography–mass spectrometry or high-performance liquid chromatography fingerprinting will enable researchers to establish chemical profiles for single herbs (e.g., catalpol or oroxylin A) and complex formulations alike. For multiherb prescriptions, consistency evaluations between different batches are crucial to ensure stable synergistic pharmacological effects. Implementing these measures, along with the integration of pharmacokinetic data to verify blood–brain barrier penetration, will transform the current preliminary and hypothesis-generating evidence into a more reliable and actionable roadmap for clinical ADHD management.

### 3.8 Study limitations

Our review has certain methodological limitations. Specifically, although we initially prioritized PubMed and Web of Science because of their extensive indexing of biomedical and pharmacological studies, we acknowledge that the exclusion of other databases may limit the comprehensiveness of our search. Nevertheless, supplemental cross-referencing suggests that the current 25 studies represent the primary body of evidence in this field. To enhance comprehensiveness and further minimize the risk of publication bias, future systematic reviews should expand the search scope to include additional major databases, such as Scopus, Embase, and the Cochrane Library. Integrating these diverse sources will help strengthen the evidence base and ensure a more exhaustive evaluation of herbal interventions for ADHD.

## 4 Conclusion

Herbal medicines for ADHD show therapeutic potential through multi-target mechanisms, including catecholaminergic modulation and suppression of neuroinflammatory signaling. However, the current preclinical evidence remains constrained by the narrow range of animal models, the heavy reliance on hyperactivity-focused behavioral tests, insufficient safety evaluation, and limited methodological rigor. Some formulations, including LMQXM and ASDZL have shown promising effects and have been discussed in relation to multiple ADHD-relevant pathways, but the overall evidence base remains preliminary. Future research should therefore prioritize more diverse ADHD models, broader behavioral assessments of attention and impulsivity, and direct comparisons with established standard therapies. Stronger standardization and systematic safety evaluation will also be essential to support more reliable clinical translation.

## Data Availability

The original contributions presented in the study are included in the article/**Supplementary Material**. Further inquiries can be directed to the corresponding author.
