# Supplementary figures and images for: Systematic review of animal studies on the use of herbal medicine for attention-deficit/hyperactivity disorder
Source: Front Psychiatry. 2026 Jun 18;17:1812398. doi: 10.3389/fpsyt.2026.1812398 (PMC13323485; doi:10.3389/fpsyt.2026.1812398)

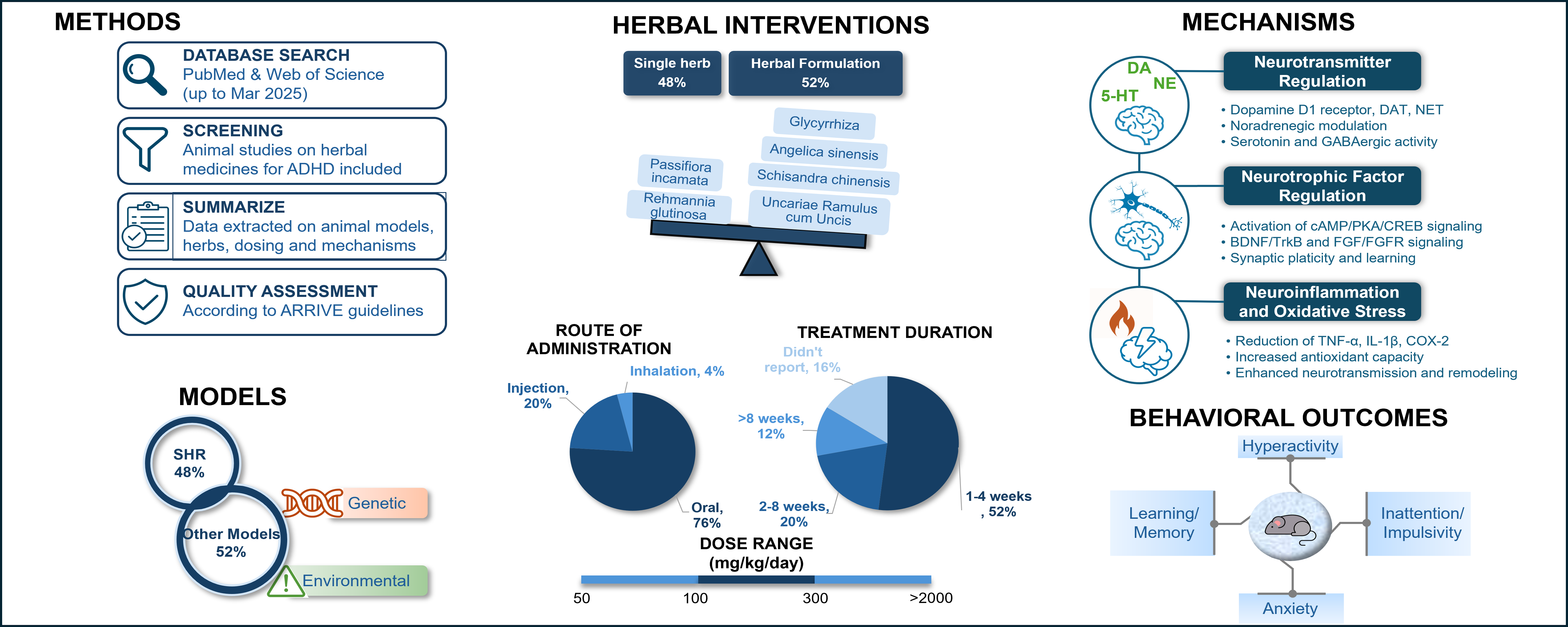

Supplement: Supplementary file 1 [file Image1.tif]
